# Secretogranin II impairs tumor growth and angiogenesis by promoting degradation of hypoxia‐inducible factor‐1α in colorectal cancer

**DOI:** 10.1002/1878-0261.13044

**Published:** 2021-07-26

**Authors:** Chao Fang, Lei Dai, Cun Wang, Chuanwen Fan, Yongyang Yu, Lie Yang, Hongxin Deng, Zongguang Zhou

**Affiliations:** ^1^ Institute of Digestive Surgery West China Hospital, Sichuan University Chengdu China; ^2^ Department of Gastrointestinal Surgery West China Hospital West China School of Medicine Sichuan University Chengdu China; ^3^ State Key Laboratory of Biotherapy and Cancer Center West China Hospital Sichuan University and Collaborative Innovation Center for Biotherapy Chengdu China

**Keywords:** angiogenesis, colorectal cancer, hypoxia‐inducible factor, secretogranin II, vascular endothelial growth factor

## Abstract

Distant metastasis is a major cause of death in patients with colorectal cancer (CRC) but the management of advanced and metastatic CRC still remains problematic due to the distinct molecular alterations during tumor progression. Tumor angiogenesis is a key step in tumor growth, invasion and metastasis. However, the signaling pathways involved in angiogenesis are poorly understood. The results of the present study showed that secretogranin II (SCG2) was significantly downregulated in malignant CRC tissues, and higher expression of SCG2 was correlated with longer disease‐free survival and overall survival of CRC patients. The results of an animal study showed that ectopic expression of SCG2 significantly inhibited CRC tumor growth by disrupting angiogenesis. Furthermore, the inhibition of expression of vascular endothelial growth factor (VEGF) by SCG2 and rescue of VEGF effectively blocked SCG2‐induced inhibition of angiogenesis. Investigations into the underlying mechanism suggested that SCG2 promoted degradation of hypoxia‐inducible factor (HIF)‐1α by interacting with the von Hippel–Lindau tumor suppressor in CRC cells. Blocking of degradation of HIF‐1α effectively attenuated the SCG2‐mediated decrease in expression of VEGF in CRC cells. Collectively, these results demonstrated that treatment with SCG2 effectively inhibited CRC tumor growth by disrupting the activities of HIF‐1α/VEGF, thereby clarifying the anti‐tumor and anti‐angiogenesis roles of SCG2 in CRC, while providing a novel therapeutic target and a potential prognostic marker of disease progression.

## Graphical abstract

Tumor angiogenesis is a key step in tumor growth, invasion and metastasis. However, the signaling pathways involved in colorectal cancer (CRC) angiogenesis are poorly understood. Here we show that secretogranin II (SCG2) was significantly downregulated in malignant CRC tissues and higher expression of SCG2 correlated with longer disease‐free survival and overall survival of patients with CRC. SCG2 impaired tumor growth and angiogenesis by inhibiting vascular endothelial growth factor expression and promoting hypoxia‐inducible factor‐1α degradation. Therefore, SCG2 may serve as a novel therapeutic target and a potential prognostic marker of disease progression in patients with CRC.

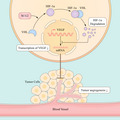

**Abbreviations**
CRC: colorectal cancer
DMEM: Dulbecco’s modified Eagle’s medium
H&E: hematoxylin and eosin
HIEC: human intestinal epithelial cell
HIF: hypoxia‐inducible factor
HVEC: human umbilical vein endothelial cells
IHC: immunohistochemistry
mRNA: messenger RNA
MOI: multiplicity of infection
SCG2: secretogranin II
VEGF: vascular endothelial growth factor
VHL: von Hippel–Lindau

## 1 Introduction

Colorectal cancer (CRC) is the third most commonly diagnosed cancers and the fourth most common cause of cancer‐related death worldwide [1]. Owing to the introduction and dissemination of screening tests, the identification of risk factors, and improvements in treatment regimens, the oncologic outcomes of CRC patients have been greatly improved over the past few decades [2, 3, 4]. However, distant metastasis, which occurs in more than 60% of CRC cases during the disease progression, is the primary cause of the death of CRC patients [5, 6, 7]. Hence, tumor metastasis is among the most critical prognostic factors for CRC and a major obstacle to successful treatment [1, 7]. The management of advanced and metastatic CRC still remains problematic due to the distinct molecular alterations during tumor progression [8, 9]. Thus, a better understanding of the molecular mechanisms underlying CRC disease progression and metastasis is needed to improve therapeutic strategies and outcomes.

The disease progression and distant metastasis of solid tumors depend on the formation of tumor‐related vessels to obtain the necessary nutrients and oxygen [10]. More specifically, angiogenesis and vasculogenesis are crucial for tumor growth, invasion and metastasis [10]; angiogenesis refers the ‘sprouting’ of new blood vessels from existing vessels, and vasculogenesis refers to the regeneration of endothelial cells from endothelial progenitor cells derived from bone marrow [11, 12]. The dependence of solid tumors on the formation of new vessels has led to the development and application of various anti‐angiogenesis agents such as bevacizumab, a monoclonal antibody against vascular endothelial growth factor (VEGF), which has improved the oncologic outcome of patients with metastatic and advanced CRC [13]. However, patients with some specific genetic types are at risk of therapeutic resistance and rapid disease progression [14, 15]. Thus, it is necessary to identify the signaling networks associated with angiogenesis and vasculogenesis to elucidate the molecular mechanisms underlying tumor invasion and metastasis.

Secretogranin II (SCG2), a key AP‐1‐regulated member of the granin family, is a neuroendocrine protein that regulates the biogenesis of secretory granules involved in hormonal peptide sorting [16], mediates neuronal differentiation, and protects neuroblastoma cells from nitric oxide‐induced apoptosis. SCG2 is cooperatively activated by glutamate‐dopamine hippocampal neurons as a potential regulator of food intake and non‐chronic bodyweight change by interacting with secretogranin III and accumulating appetite‐related hormones into secretory granules [17]. Various studies have demonstrated pro‐angiogenesis roles of SCG2 in the process of wound injury [18] and ovulation [19] based on the direct effects in endothelial cells. In addition, SCG2 has been implicated as a candidate tumor suppressor in several tumors [20]. SCG2 expression has also been associated with the survival outcome of CRC patients [21, 22]. However, the functional significance of SCG2 in CRC still remains unclear.

Therefore, the aim of the present study was to investigate the expression and function of SCG2 in CRC. The protein expression profile of SCG2 in CRC was determined in 270 pairs of malignant and adjacent normal tissues, followed by correlation analysis of the prognostic outcomes of CRC patients. The function of SCG2 was determined with the use of a mouse xenograft model and the underlying mechanism was investigated by RNA sequencing, co‐immunoprecipitation and other technologies. The study indicated a role of SCG2 in the disease progression of CRC, thereby providing a potential therapeutic target and a predictor of the survival of CRC patients.

## 2 Materials and methods

### 2.1 Clinical sample

This study enrolled CRC patients at the Department of Gastrointestinal Surgery, West China Hospital, Sichuan University. All the tumor and paired adjacent normal tissues were collected from CRC patients undergoing curative resection between 2013 and 2014. All the frozen tissues were fixed with 4% paraformaldehyde, embedded in paraffin and cut into 4‐μm slides for hematoxylin and eosin (H&E) staining. The histopathological evaluation was performed by one experienced pathologist and reviewed by other two pathologists. The qualified samples were used for tissue microarray by Outdo Biotech (Shanghai, China); the tissue microarray was named the HXCRC (HuaXi Colorectal Cancer) cohort. All patients were suggested for postoperative treatment and scheduled for periodic follow‐ups as recommend by NCCN guidelines. The disease‐free survival and overall survival were recorded by arranging follow‐up in the next 5 years after surgery. The study methodologies conformed to the standards set by the Declaration of Helsinki. Informed consent was collected from all patients and the experiments were approved by the Research Ethics Committee of West China Hospital, Sichuan University [Number: 2017(169)].

### 2.2 Cell culture and treatment

Human CRC cells SW620, SW480, RKO, HCT116, LoVo, DLD‐1, Caco‐2, HT‐29 and normal human intestinal epithelial cell (HIEC) were purchased from American Type Culture Collection (ATCC, Manassas, VA, USA). SW620, SW480, RKO, HCT116, DLD‐1, Caco‐2, HT‐29 and normal HIEC were cultured in Dulbecco’s modified Eagle’s medium (DMEM; Thermo Fisher Scientific, Inc., Waltham, MA, USA) supplemented with 10% FBS (Thermo Fisher Scientific, Inc.) and LoVo cells were cultured in Minimum Essential Medium (Thermo Fisher Scientific, Inc.) supplemented with 10% FBS (Thermo Fisher Scientific, Inc.). Human umbilical vein endothelial cells (HUVEC) were obtained from human umbilical veins as previously described and cultured in EGM medium (Merck Millipore, Burlington, MA, USA). All cells were incubated at 37°C with 5% CO_2_. Lentivirus‐based SCG2 overexpression system and empty vector control were purchased from Gene Pharma (Shanghai, China) and used to infect SW620 and RKO cells [multiplicity of infection (MOI) = 20]. At 24 h postlentivirus infection, the culture supernatant was removed and new medium containing 2 mg·mL^−1^ puromycin was added to select stable‐infected cells.

### 2.3 Western blotting

SW620 and RKO cells were collected and lysed in RIPA lysis buffer (Beyotime, Beijing, China) containing protease inhibitor cocktail (Merck Millipore). After centrifugation for 12 000 ***g*** at 4°C for 15 min, the protein was collected for concentration determination by BCA assay (Thermo Fisher Scientific, Inc.). A total of 10 µg proteins was loaded per lane, separated by 10% and 7.5% SDS/PAGE and transferred to 0.45‐µm polyvinylidene fluoride membranes (Merck Millipore). After blocking with TBS‐Tween‐20 (0.1% Tween‐20) containing 0.5% non‐fat milk, the membranes were incubated with primary antibodies at 4°C overnight. Following incubation with anti‐mouse IgG or anti‐rabbit IgG secondary antibodies at 37°C for 1 h, protein expression was detected by chemiluminescence with a kit from Merck Millipore.

### 2.4 Animal study

Female BALB/c nude mice (5–6 weeks old) were purchased from HFKbio (Beijing, China) and allowed to acclimate for 1 week before use. All mice were given free access to food and water in a specific pathogen‐free condition. SW620 and RKO cells that were stably infected with lenti‐SCG2 or lenti‐control (5 × 10^6^ cells) were injected into the flank of mice to establish a subcutaneous cancer model. Tumor length and width were measured by V caliper every 3 days and the tumor volume was calculated as length × width^2^ × 0.52. At 22 days postcell injection, mice were sacrificed and tumors were collected, weighed and used for further analysis. For Matrigel plug assay, 2 × 10^6^ cells embedded in 100 µL Matrigel were injected into the flank of mice. Seven days later, the Matrigel plug was collected and used for further analysis. Human SCG2 protein and control IgG were purchased from ProteinTech (Wuhan, China) and were injected into the tumors at 8, 10 and 12 days post‐SW620 cell injection. The entire animal study was approved by the Institutional Animal Care and Use Committees of Sichuan University.

### 2.5 Histological staining

Tumor tissue sections (5 μm thick) and CRC tissue microarray were deparaffinized with xylene, rehydrated in a graded ethanol series, and washed in ultrapure water. After autoclaving at 120°C for 5 min for antigen retrieval with citrate repair buffer (pH 6.0; Origene, Beijing, China), the sections were incubated in 3% H_2_O_2_ and 5% goat serum at room temperature for 15 min, and incubated with primary antibodies against SCG2 (LS‐B10869; Life Span Biosciences, Seattle, WA, USA), hypoxia‐inducible factor (HIF)‐1α (ab51608; Abcam, Cambridge, UK), VEGF (ab150375; Abcam) and CD31 (ab28364; Abcam) overnight at 4°C. For immunohistochemical staining, the sections were incubated with secondary antibodies (Zsbio, Beijing, China) and HRP‐conjugated streptomycin solution (Zsbio) for 30 min at 37°C and stained with 3, 3‐diaminobenzidine (Maixin, Fuzhou, China). For immunofluorescence staining, the sections were incubated with Texas Red‐conjugated secondary antibody (Thermo Fisher Scientific, Inc.) at 37°C for 1 h and cell nucleus was stained with 4′,6‐diamidino‐2‐phenylindole (DAPI, Thermo Fisher Scientific, Inc.). The pictures were taken with a BX 51 microscope (Olympus, Tokyo, Japan).

### 2.6 HUVEC tube formation assay and invasion assay

For tube formation, 2 × 10^4^ HUVEC in 100 µL of conditional medium (cell culture supernatant) were seeded into a 96‐well containing 50 µL Matrigel (Merck Millipore) and incubated at 37°C with 5% CO_2_ for 4 h. The tube photos were taken by inverted microscope and branch points in each frame were counted. For invasion assay, 50 µL Matrigel (Merck Millipore) diluted with DMEM (1 : 5 dilution) was added into a Transwell insert (8.0 µm; Corning, Kennebunk, ME, USA) and 2 × 10^4^ HUVEC in 100 µL of conditional medium (cell culture supernatant) was seeded into the coated Transwell. After 24 h of incubation, non‐invaded cells were removed with a cotton swab from the upper part of the Transwell and inserts were fixed with 4% paraformaldehyde for 15 min at room temperature. The inserts were stained in 500 µL of 0.03% crystal violet solution (Beyotime) and sheeted with neutral balsam (Beyotime). Five pictures were taken for each insert and the number of invaded cells in each frame was counted.

### 2.7 RNA extraction and analysis

SW620 and RKO cells and tumors were collected for RNA extraction using Trizol (Invitrogen, Waltham, MA, USA), following the manufacturer’s instructions. RNA concentration was measured by ND2000 (Thermo Fisher Scientific, Inc.) and the quality and quantity were determined using a NanoPhotometer® spectrophotometer (Implen, Munich, Germany), RNA Nano 6000 Assay Kit and the Bioanalyzer 2100 system (Agilent, Santa Clara, CA, USA). For quantitative RT‐PCR, 1 µg RNA was converted to cDNA using PrimeScript RT reagent Kit with gDNA Eraser (RR047B, Takara, Tokyo, Japan). TB green Premix EX TaqII (RR820A, Takara, Tokyo, Japan) was used for quantitative PCR reactions in CF96 (Bio‐Rad, Hercules, CA, USA). Relative VEGF expression was determined by $2^{‐\Delta \Delta C_{t}}$, where *C*_t_ is the mean threshold cycle difference after normalization to GAPDH. RNA sequencing was performed by Chengdu Base Biotech Co., Ltd (Chengdu, China) as previously reported [23]. Significantly differentially expressed messenger RNA (mRNA) was screened based on absolute value of log2 (fold change) ≥ 1, at a *P*‐value < 0.05. KEGG pathway enrichment was analyzed using the ‘ggplot2’ r package and the online biological tool, kobas 3.0 (http://kobas.cbi.pku.edu.cn).

### 2.8 ELISA

The cell culture supernatants of SW620 and RKO cells were collected and stored at −80°C. VEGF expression was determined by ELISA kit according to the manufacturer’s instructions (NeoBioscience, Shenzhen, China).

### 2.9 Co‐immunoprecipitation

SW620 and RKO cells were collected and lysed with RIPA lysis buffer (Beyotime) containing 1% protease inhibitor cocktail (Merck Millipore). After centrifugation for 12 000 ***g*** at 4°C for 15 min, the protein was collected for concentration determination by BCA assay (Thermo Fisher Scientific, Inc.). Co‐immunoprecipitation was performed according to the instructions of PureProteome™ Protein A and Protein G MagneticBeads (Merck Millipore). A total of 500 µL protein was added for the direct immunoprecipitation, and 10 µL primary antibodies against SCG2 or von Hippel‐Lindau (VHL) were added to immunoprecipitate the specific proteins. The immunoprecipitated products were collected for western blotting.

### 2.10 Statistical analysis

Continuous variables are presented as the mean ± standard deviation. Explorative comparisons of groups were using the *t*‐test for normally distributed data, and the Kruskal–Wallis test or the Mann–Whitney *U*‐test for non‐normally distributed data. Pearson’s χ^2^‐test was used to perform the distribution of nominal‐ or ordinal‐scaled variables. The Kaplan–Meier method and the log‐rank (Mantel–Cox) test were used to investigate the time‐dependent survival probabilities to compare different subgroups of CRC patients. All statistical tests were two‐sided and a probability (*P*) value of < 0.05 was considered statistically significant. All statistical analyses were performed using ibm spss statistics for Windows, version 22.0. (IBM Corporation, Armonk, NY, USA).

## 3 Results

### 3.1 Downregulation of SCG2 is correlated with poor prognosis of CRC patients

**Fig. 1:**
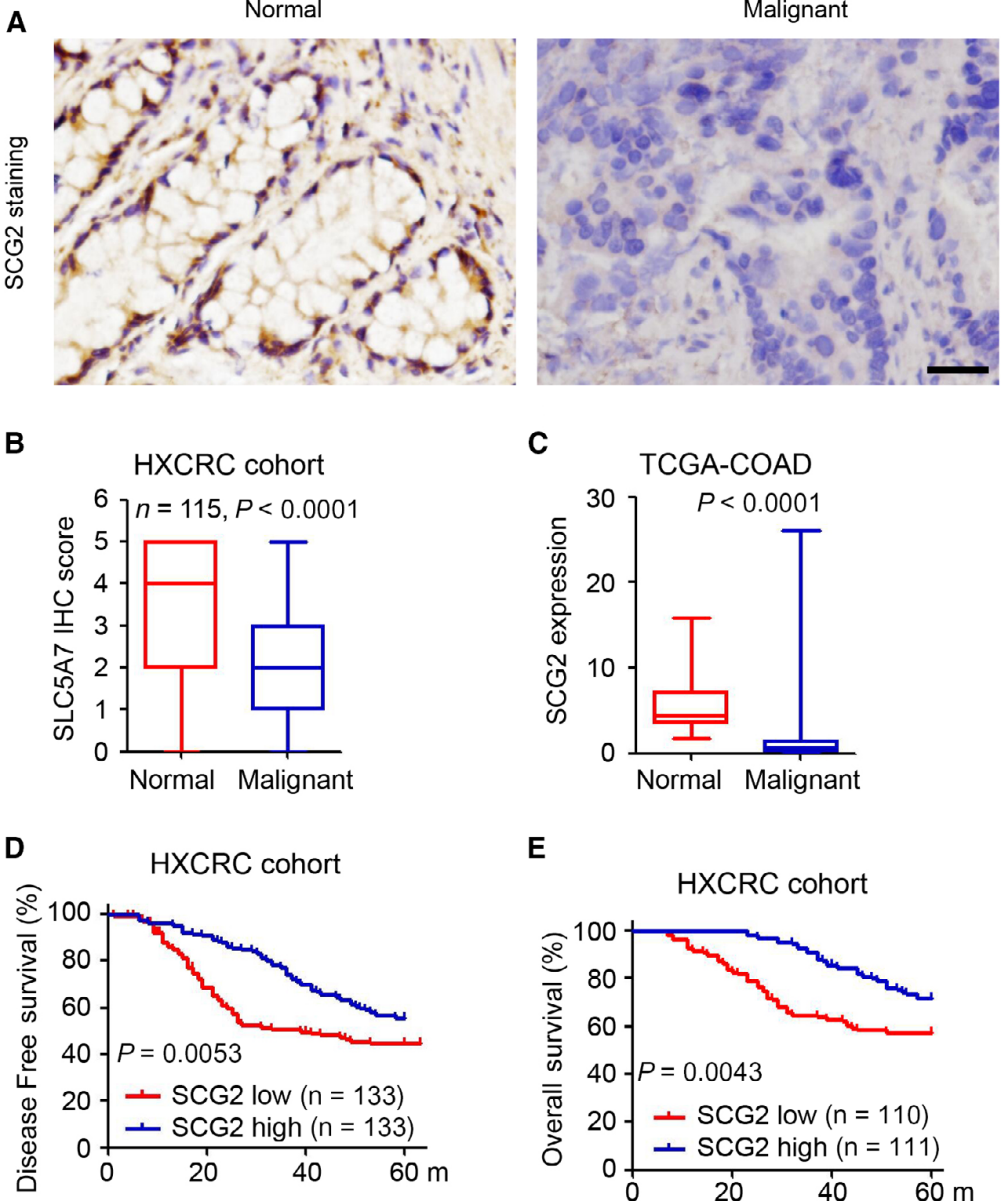
Downregulation of SCG2 is correlated with poor prognosis of CRC patients. (A) IHC staining of SCG2 expression in CRC tissues and normal colorectal tissues (*n* = 115). Scale bar: 100 μm. (B) Analysis of SCG2 expression in 115 paired CRC tissues (*t*‐test). (C) Analysis of SCG2 expression in 46 normal colorectal tissues and 155 CRC malignant tissues from the TCGA‐COAD database (*t*‐test). (D, E) Log‐rank test of disease‐free survival (D) and overall survival (E) of CRC patients with high vs. low SCG2 expression based on IHC staining in the HXCRC cohort (Log‐rank test).

Immunohistochemistry (IHC) staining was employed to determine the expression patterns of SCG2 in 270 CRC tissues and 115 normal intestinal tissues. The results revealed that SCG2 was mainly expressed in the cytoplasm of normal intestinal epithelial cells with relatively fewer SCG2‐positive cells in malignant CRC tissues (Fig. 1A). Furthermore, SCG2 was significantly downregulated in malignant CRC tissues as compared with normal tissues (Fig. 1B). In accordance with the expression data retrieved from TCGA‐COAD database, SCG2 mRNA was significantly downregulated in malignant CRC tissues (Fig. 1C). The patients in the HXCRC cohort who were further divided into low and high SCG2 expression; the latter achieved longer disease‐free and overall survival (Fig. 1D,E). These results demonstrated that downregulation of SCG2 is correlated with a poor prognosis of CRC patients.

### 3.2 SCG2 impairs CRC tumor growth

**Fig. 2:**
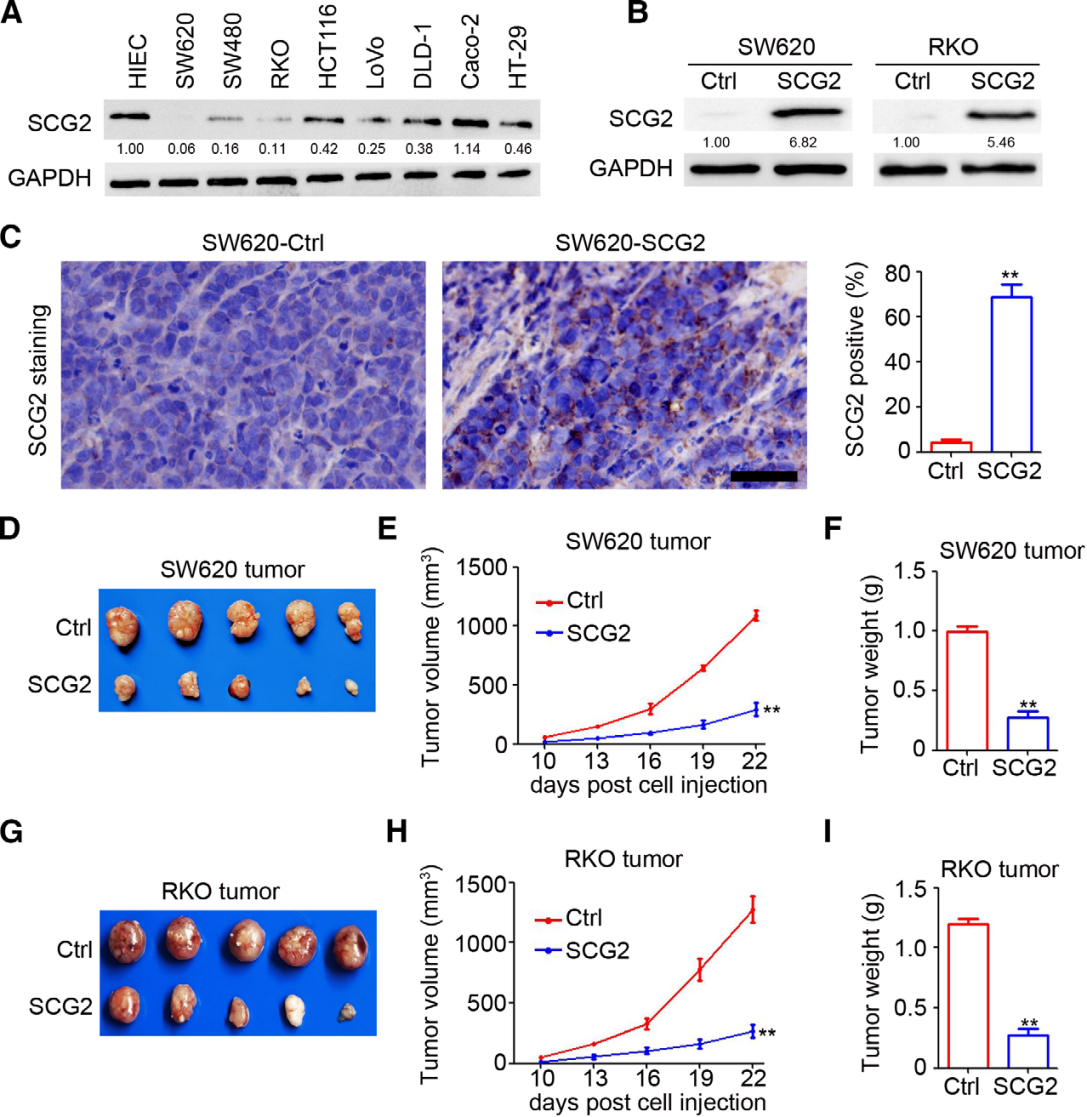
SCG2 impairs CRC tumor growth. (A) Proteins were extracted from CRC cells and HIEC for western blot analysis. (B) SW620 and RKO cells were stably infected with lenti‐control (Ctrl) and an SCG2 overexpression system. Proteins were extracted from SW620‐Ctrl, SW620‐SCG2, RKO‐Ctrl, and RKO‐SCG2 cells for western blot analysis. (C) IHC staining of SCG2 expression in SW620‐Ctrl and SW620‐SCG2 tumors. Scale bar: 100 μm. Analysis of SCG2‐positive cells in SW620 tumors (*n* = 4, **P < 0.01, mean ± SD, *t*‐test). (D) Images of SW620‐Ctrl and SW620‐SCG2 tumors. (E) By 10 days postinjection, the SW620‐Ctrl and SW620‐SCG2 tumor length and width were measured every 3 days and a tumor growth curve was generated (*n* = 5, **P < 0.01, mean ± SD, *t*‐test). (F) Analysis of SW620‐Ctrl and SW620‐SCG2 tumor weights (*n* = 5, **P < 0.01, mean ± SD, *t*‐test). (G) Images of RKO‐Ctrl and RKO‐SCG2 tumors. (H) By 10 days postinjection, the RKO‐Ctrl and RKO‐SCG2 tumor length and width were measured every 3 days and a tumor growth curve was generated (*n* = 5, **P < 0.01, mean ± SD, *t*‐test). (I) Analysis of RKO‐Ctrl and RKO‐SCG2 tumor weights (*n* = 5, **P < 0.01, mean ± SD, *t*‐test).

A lentivirus‐based SCG2 overexpression system was employed to investigate the potential function of SCG2 in CRC. As shown in Fig. 2A, SCG2 expression was decreased in most CRC cell lines, with the exception of Caco‐2 cells. After stable infection with lenti‐SCG2, ectopic expression of SCG2 was demonstrated in SW620‐SCG2 and RKO‐SCG2 cells (Fig. 2B). Further IHC staining also indicated that SCG2 was significantly upregulated in SW620‐SCG2 tumors (Fig. 2C). In the subcutaneous xenograft model, ectopic expression of SCG2 dramatically inhibited the growth of SW620 tumor cells by 73.0% in tumor volume as compared with control cells (293.1 ± 125.6 vs. 1086.4 ± 94.8 mm^3^, respectively, Fig. 2D,E) and by 72.7% in tumor weight (0.27 ± 0.13 vs. 0.99 ± 0.10 g, respectively, Fig. 2F). Similar inhibition was also confirmed in the growth of RKO tumor cells in mice as compared with the controls by volume (267.2 ± 122.0 vs. 1275.9 ± 241.8 mm^3^, respectively, Fig. 2G,H) and weight (0.27 ± 0.13 vs. 1.20 ± 0.10 g, respectively, Fig. 2I). These results demonstrated the anti‐tumor effects of SCG2 in CRC.

### 3.3 SCG2 inhibits angiogenesis in CRC

**Fig. 3:**
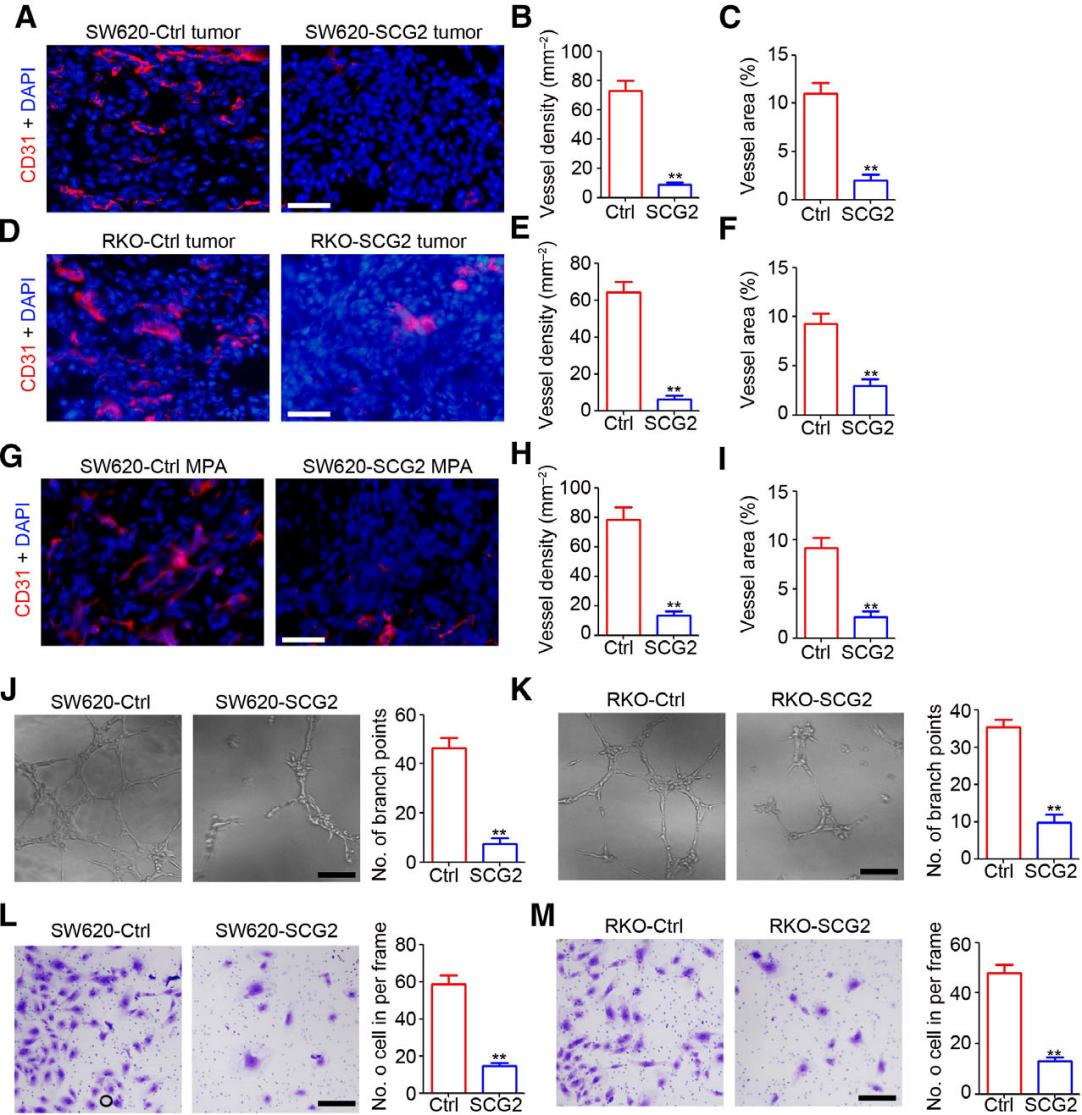
SCG2 inhibits angiogenesis in CRC. (A) IHC staining of CD31 in SW620‐Ctrl and SW620‐SCG2 tumors (*n* = 4). Scale bar: 100 μm. (B, C) Analysis of vessel density (B) and area (C) in SW620‐Ctrl and SW620‐SCG2 tumors (*n* = 4, **P < 0.01, mean ± SD, *t*‐test). (D) IHC staining of CD31 in RKO‐Ctrl and RKO‐SCG2 tumors (*n* = 4). Scale bar: 100 μm. (E, F) Analysis of vessel density (E) and area (F) in RKO‐Ctrl and RKO‐SCG2 tumors (*n* = 4, **P < 0.01, mean ± SD, *t*‐test). (G) IHC staining of CD31 in SW620‐Ctrl and SW620‐SCG2 Matrigel‐based plug assay (MPA) (*n* = 4). Scale bar: 100 μm. (H, I) Analysis of vessel density (H) and area (I) in SW620‐Ctrl and SW620‐SCG2 MPA (*n* = 4, **P < 0.01, mean ± SD, *t*‐test). (J, K) The supernatants of the indicated cultured cells were collected and used as conditioned medium for HUVEC tube formation. The branch points per frame were counted and analyzed (*n* = 4, **P < 0.01, mean ± SD, *t*‐test). Scale bar: 100 μm. (L, M) The supernatant of the indicated cultured cells was collected and used as conditioned medium for the HUVEC invasion assay. The invaded cells per frame were counted and analyzed (*n* = 4, **P < 0.01, mean ± SD *t*‐test). Scale bar: 100 μm.

Next, the potential regulatory role of SCG2 in tumor angiogenesis was investigated by IHC staining of CD31. As shown in Fig. 3A, there were fewer CD31‐positive cells in the SW620‐SCG2 group than the SW620‐Ctrl group. Further analysis confirmed the inhibition of vessel density (Fig. 3B) and vessel area (Fig. 3C) by SCG2 in SW620 tumors. Similar results were also demonstrated in RKO tumors (Fig. 3D), as evidenced by significant inhibition of vessel density and area (Fig. 3E,F, respectively). Furthermore, the results of the Matrigel‐based plug assay showed that ectopic SCG2 expression significantly inhibited angiogenesis in the SW620‐SCG2 group as compared with the SW620‐Ctrl group (Fig. 3G–I). Cell culture supernatant was collected for a HUVEC‐based tube formation assay and invasion assay. As shown in Fig. 3J, the supernatant of the SW620‐SCG2 cell culture significantly inhibited tube formation of HUVEC. Meanwhile, less tube formation was observed in the HUVEC treated with RKO‐SCG2 conditioned medium (Fig. 3H). The results of the HUVEC‐based invasion assay showed that the supernatant of the SW620‐SCG2 and RKO‐SCG2 cell cultures also dramatically decreased the invasion ability of HUVEC (Fig. 3L,M). Collectively, ectopic expression SCG2 in CRC cells significantly inhibited tumor angiogenesis.

### 3.4 SCG2 inhibits angiogenesis via downregulating expression of VEGF

**Fig. 4:**
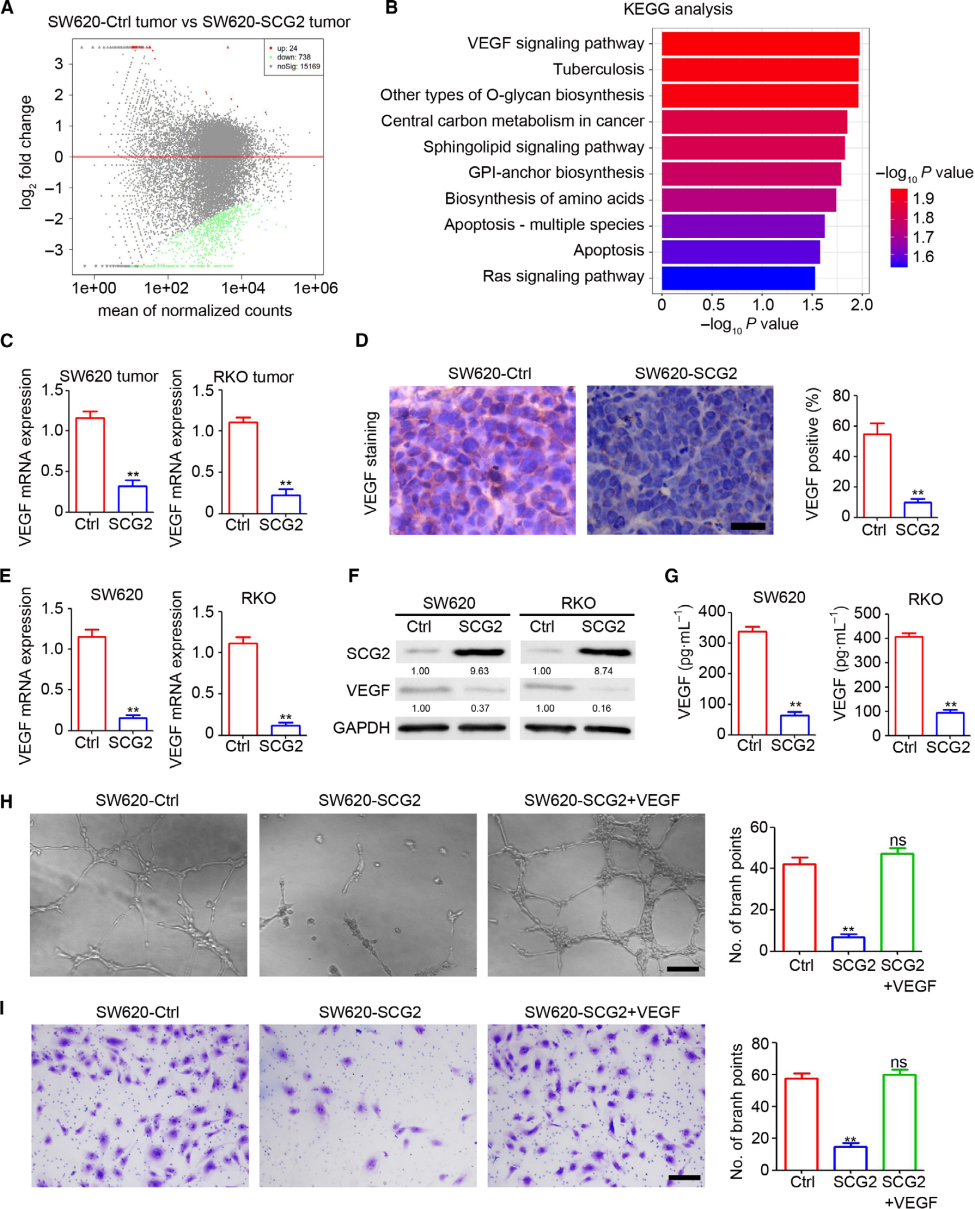
SCG2 inhibits angiogenesis via downregulating VEGF expression. (A) Sequencing of coding RNA in SW620‐Ctrl and SW620‐SCG2 tumors. (B) KEGG analysis of deregulated coding RNA between SW620‐Ctrl and SW620‐SCG2 tumors. (C) qPCR analysis of VEGF mRNA expression in the indicated tumors (*n* = 3, **P < 0.01, mean ± SD, *t*‐test). (D) IHC staining of VEGF expression in SW620‐Ctrl and SW620‐SCG2 tumors. Scale bar: 100 μm. Analysis of VEGF‐positive cells in SW620 tumors (*n* = 4, **P < 0.01, mean ± SD, *t*‐test). (E) qPCR analysis of VEGF mRNA expression in the indicated cells (*n* = 3, **P < 0.01, mean ± SD, *t*‐test). (F) Proteins of the indicated cells were extracted for western blot analysis. (G) The cell culture supernatants of indicated cells were collected for use in an ELISA (*n* = 3, **P < 0.01, mean ± SD, *t*‐test). (H, I) VEGF protein was added to the cell culture supernatant of SW620‐SCG2 cells to rescue VEGF expression. The conditioned medium was used for the HUVEC tube formation assay (H) and invasion assay (I). Scale bar: 100 μm (*n* = 3, **P < 0.01; ns, no significant difference, mean ± SD, *t*‐test).

To investigate the molecular mechanisms underlying inhibition of tumor angiogenesis by SCG2 in CRC, mRNA sequencing was employed to determine the expression profiles of coding RNA in SW620 tumors. As shown in Fig. 4A, 738 genes were downregulated and 24 genes upregulated in SW620‐SCG2 tumors as compared with SW620‐Ctrl tumors. KEGG analysis indicated that the deregulated genes in the VEGF signaling pathway played crucial roles in angiogenesis (Fig. 4B). Furthermore, the qPCR results confirmed the inhibition of VEGF‐165 by SCG2 in SW620 and RKO tumors (Fig. 4C). Also, fewer VEGF‐positive cells were observed in SW620‐SCG2 tumors (Fig. 4D) and ectopic expression of SCG2 inhibited transcription of VEGF mRNA (Fig. 4E), resulting in lower expression of intracellular and excretive VEGF protein (Fig. 4F,G, respectively). To determine whether VEGF plays a crucial role in the inhibition of tumor angiogenesis by SCG2, a HUVEC‐related assay was performed after rescuing expression of VEGF in the supernatant of SW620‐SCG2 cells. The results revealed that rescuing VEGF expression efficiently blocked SCG2‐mediated inhibition of HUVEC‐related tube formation (Fig. 4H) and invasion (Fig. 4I). These results demonstrated that SCG2 inhibited tumor angiogenesis via decreasing expression of VEGF in CRC cells.

### 3.5 SCG2 promotes degradation of HIF‐1α via binding to VHL

**Fig. 5:**
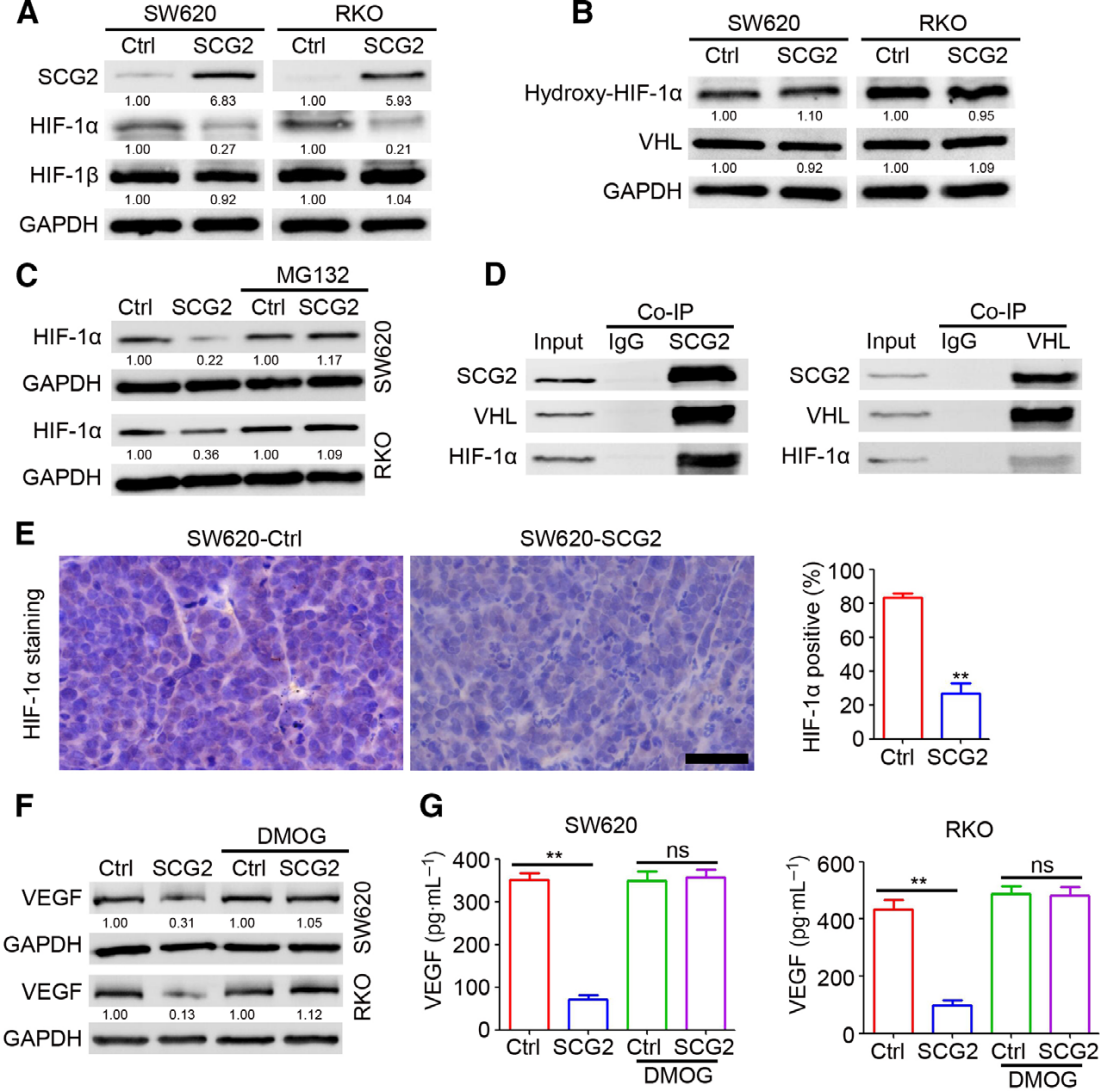
SCG2 promotes HIF‐1α degradation via binding to VHL. (A, B) Proteins of the indicated cells were extracted for western blot analysis. (C) The indicated cells were treated with MG132. After 4 h, proteins were extracted for western blot analysis. (D) Proteins of SW620‐SCG2 cells were extracted for co‐immunoprecipitation analysis. The immunoprecipitated proteins were used for western blot analysis. (E) IHC staining of HIF‐1α expression in SW620‐Ctrl and SW620‐SCG2 tumors. Scale bar: 100 μm. Analysis of HIF‐1α‐positive cells in SW620 tumors (*n* = 4, **P < 0.01, mean ± SD, *t*‐test). (F, G) The indicated cells were treated with DMOG. After 24 h, proteins were extracted for western blot analysis (F) and the cell culture supernatant was collected for use in an ELISA (G, *n* = 3, **P < 0.01, mean ± SD, *t*‐test).

The expression of VEGF was inhibited by SCG2 at the mRNA and protein levels, indicating that SCG2 inhibits expression of VEGF at the transcriptional level. However, SCG2 is a cytoplasmic protein. Thus, we speculated that SCG2 may inhibit the transcription of VEGF by regulating the expression of transcription factors, such as HIF‐1. The results indicated that ectopic SCG2 expression inhibited the expression of HIF‐1α but had no significant effect on HIF‐1β (Figs 5A and S1A). The expression of hydroxy‐HIF‐1α and VHL was not regulated by SCG2 in SW620 and RKO cells (Fig. 5B). Inhibition of protease activity by MG132 blocked SCG2‐mediated inhibition of HIF‐1α expression in CRC cells (Fig. 5C), indicating that SCG2 promotes degradation of HIF‐1α. The results of the co‐immunoprecipitation assay suggested that SCG2 interacted with VHL and HIF‐1α in CRC cells (Figs 5D and S1B–E). Compared with the SW620‐Ctrl tumors, the SW620‐SCG2 tumors contained fewer HIF‐1α‐positive cells (Fig. 5E). Treatment of SW620‐Ctrl and SW620‐SCG2 cells with DMOG, an activator of HIF‐1α, attenuated SCG2‐mediated inhibition of VEGF expression in SW620 and RKO cells (Fig. 5F,G). These results suggested that SCG2 inhibited VEGF expression by promoting interactions with VHL and degradation of HIF‐1α.

### 3.6 Inverse correlation between SCG2 and HIF‐1α/VEGF in CRC tissues

**Fig. 6:**
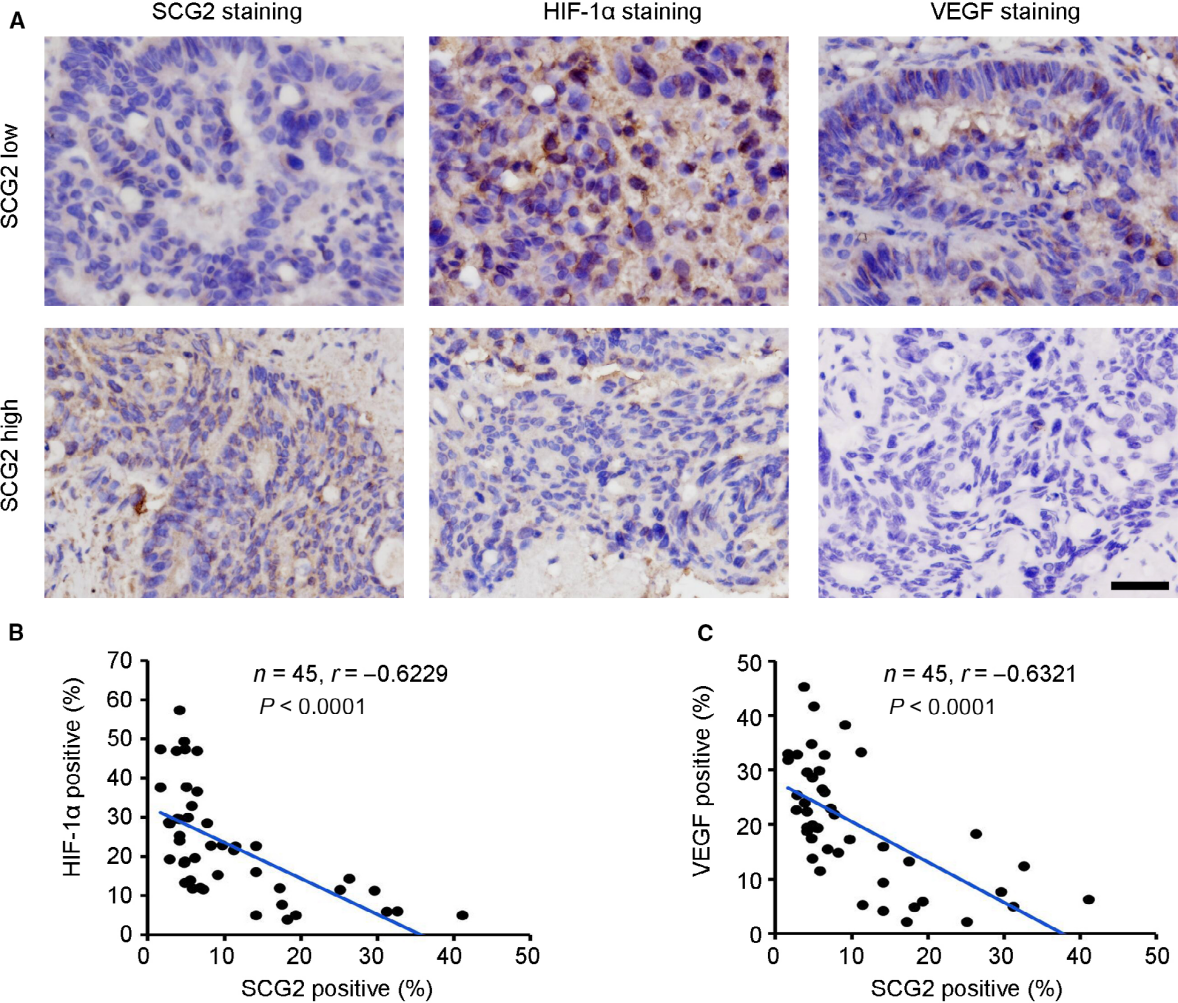
Inverse correlation between SCG2 and HIF‐1α/VEGF in CRC tissues. (A) IHC staining of SCG2, HIF‐1α and VEGF expression in 45 CRC malignant tissues. Scale bar: 100 μm. (B) Correlation analysis of SCG2‐positive cells and HIF‐1α‐positive cells in 45 CRC malignant tissues (*n* = 45, Pearson’s χ^2^‐test). (C) Correlation analysis of SCG2‐positive cells and VEGF‐positive cells in 45 CRC malignant tissues (*n* = 45, Pearson’s χ^2^‐test).

Based on the negative regulatory role of SCG2, the potential correlation with HIF‐1α/VEGF expression in CRC tissues was investigated. IHC staining was employed to determine the expression profiles of SCG2, HIF‐1α and VEGF expression in 45 malignant CRC tissues by analyzing the percentage of positive cells in each specimen. The results revealed high expression of HIF‐1α and VEGF in specimens with low SCG2 expression, and low expression of HIF‐1α and VEGF in specimens with high SCG2 expression (Fig. 6A). Correlation analysis indicated that in CRC tumor tissues, SCG2 expression was inversely correlated with HIF‐1α expression (Fig. 6B, *r* = −0.6229) as well as VEGF expression (Fig. 6C, *r* = −0.6321). These results confirmed an inverse correlation between SCG2 and HIF‐1α/VEGF expression in CRC tumor tissues.

### 3.7 SCG2 protein inhibits CRC tumor growth in mice

**Fig. 7:**
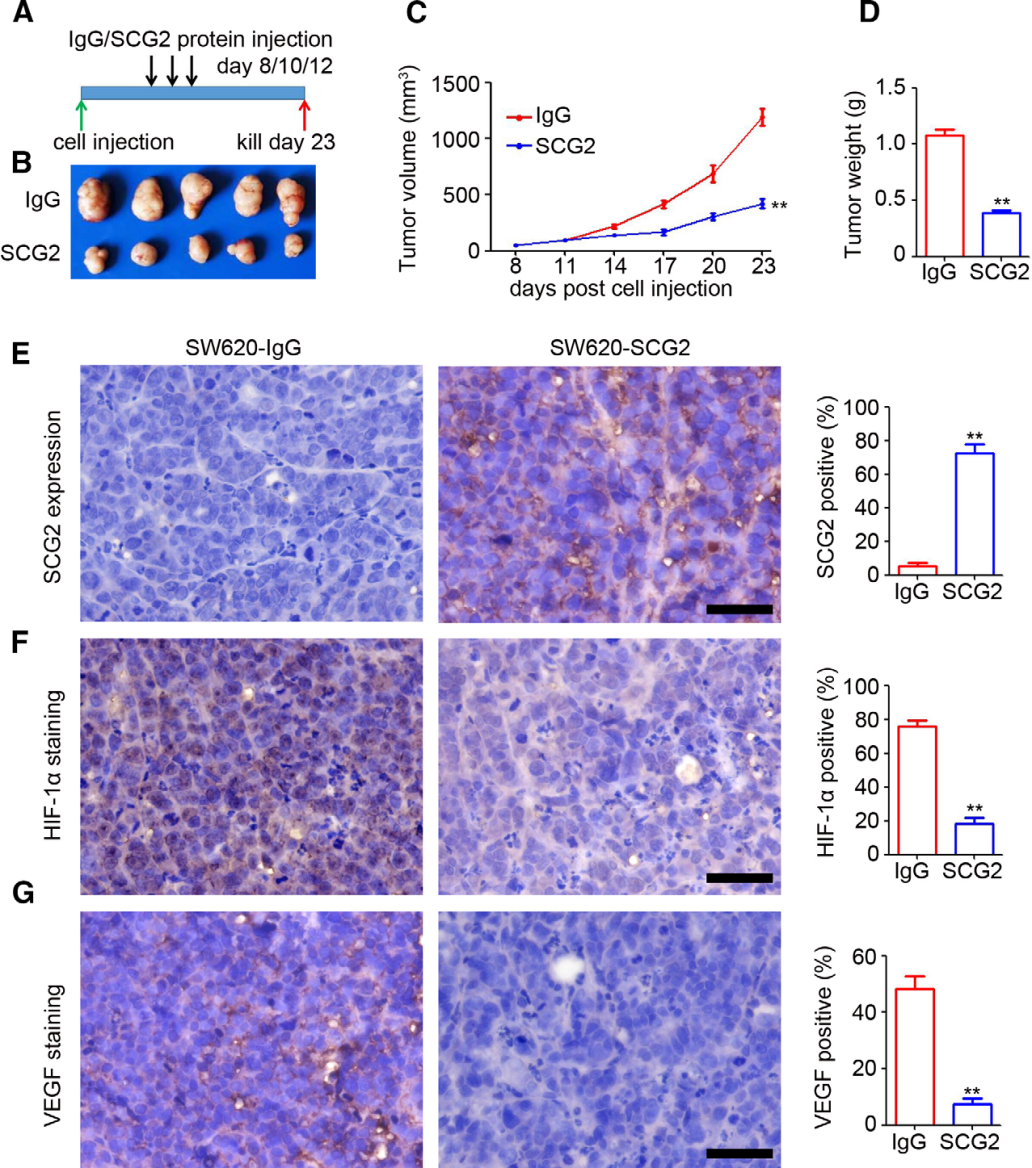
SCG2 protein inhibits CRC tumor growth in mice. (A) IgG and SCG2 (10 μg/mice/time) were injected into the tumors at 8, 10 and 12 days post‐SW620 cell injection. (B) Image of SW620 tumors treated with IgG or SCG2 protein. (C) At 8 days post‐injection, the SW620 tumor length and width were measured every 3 days and a tumor growth curve was generated (*n* = 5, **P < 0.01, mean ± SD, *t*‐test). (D) Analysis of SW620‐IgG and SW620‐SCG2 tumor weights (*n* = 5, **P < 0.01, mean ± SD, *t*‐test). IHC staining of SCG2 (E), HIF‐1α (F), and VEGF (G) expression in SW620‐IgG and SW620‐SCG2 tumors (*n* = 4). Scale bar: 100 μm. Analysis of SCG2‐, HIF‐1α‐ and VEGF‐positive cells in SW620 tumors (*n* = 4, **P < 0.01, mean ± SD, *t*‐test).

To determine the potential therapeutic effect of SCG2 on CRC tumor growth, the tumors were treated with SCG2 protein at 8, 10 and 12 days postinjection of SW620 cells (Fig. 7A). The results indicated that treatment with the SCG2 protein dramatically inhibited SW620 tumor growth as compared with the controls (IgG) by 64.7% in tumor volume (419.6 ± 91.0 vs. 1190.2 ± 163.7 mm^3^, respectively, Fig. 7B,C) and by 64.3% in tumor weight (0.38 ± 0.05 vs. 1.08 ± 0.12 g, respectively, Fig. 7D). The IHC staining results revealed that treatment with SCG2 protein increased the number of SCG2‐positive cells in SW620 tumors (Fig. 7E), accompanied by inhibition of HIF‐1α (Fig. 7F) and VEGF (Fig. 7G). These results demonstrated the therapeutic effect of the SCG2 protein in CRC.

## 4 Discussion

Tumor metastasis is a multistage process in which malignant cells disseminate from the primary tumor and colonize distant organs and/or nodes. During the metastasis process, angiogenesis and vasculogenesis are essential and critical steps for the formation of tumor‐related vessels. Thus, it is increasingly important to understand the biology of angiogenesis and vasculogenesis in different types of cancers. SCG2 is a neuroendocrine protein that is diffusely expressed in tissues of the prostate, pancreas and gastrointestinal tract, whereas in normal tissues, SCG2 expression is relatively high. SCG2 has been implicated as a pro‐angiogenesis factor in wound injury and ovulation. However, given the tissue specificity, the role of SCG2 likely differs among different tumor types. Analysis of clinical samples demonstrated that SCG2 was downregulated in CRC tumor tissues, whereas high SCG2 expression was correlated with a good prognosis of CRC patients. Ectopic SCG2 expression significantly inhibited CRC tumor growth by disrupting tumor angiogenesis. Further results confirmed that the inhibition of VEGF expression by SCG2 inhibited angiogenesis in CRC tumors and also revealed that SCG2 interacted with VHL to promote VHL‐dependent degradation of HIF‐1α and block degradation of HIF‐1α, which attenuated the SCG2‐mediated decreased expression of VEGF. These findings indicated that SCG2 had anti‐tumor and anti‐angiogenesis roles in CRC during disease progression.

Secretogranin II is widely expressed in diverse organs and tissues, and is deregulated in various cancers [24]. The results of the present study revealed that expression of SCG2 was significantly downregulated at both protein and mRNA levels in malignant CRC tissues, but highly expressed in paired adjacent normal tissues. Some previous studies have reported significantly poorer survival of CRC patients with high expression signatures of five genes, including SCG2, as compared with low‐risk patients [22]. The expression of stroma‐related SCG2 was also significantly associated with poorer survival of CRC patients [21]. In the present study, SCG2 protein expression was determined in 270 malignant tissues of CRC patients who were divided into two groups based on expression levels of SCG2 protein in tumor cells, but not in stromal cells. Prognosis analysis indicated that higher expression of SCG2 in tumor cells was correlated with longer disease‐free and overall survival, which is inconsistent with the results of previous studies [21, 22]. This discrepancy may have resulted from differences in the analytical approaches. Specifically, in the present study, expression of SCG2 protein was quantified in tumor cells, whereas in the cited previous studies, expression was measured in total cells. The results of the present study suggest that expression of SCG2 protein in tumor cells is a positive prognostic predictor of CRC patients.

Secretogranin II, as a key AP‐1‐regulated protein, mediates neuronal differentiation and protects neuroblastoma cells from nitric oxide‐induced apoptosis [25]. When co‐activated by glutamate‐dopamine hippocampal neurons, SCG2 is a potential regular of food intake and non‐chronic bodyweight change by interaction with secretogranin III and accumulating appetite‐related hormones into secretory granules [17]. In this study, the potential function of SCG2 in CRC tumor growth was investigated. The results showed that ectopic expression of SCG2 significantly inhibited CRC tumor growth in mice. Treatment with SCG2 protein had a therapeutic effect on CRC tumor growth. However, further studies are needed to determine the potential therapeutic effects on patient‐derived in addition to CRC cell‐based xenograft.

The results of the present study demonstrated an inhibitory role of SCG2 in tumor angiogenesis, which is inconsistent with the findings of previous studies [18, 19]. In both diabetic and burn wounds after fasting, expression of SCG2 was increased in endothelial cells [18]. Knockdown of SCG2 in endothelial cells markedly attenuated the fasting/refeeding‐induced pro‐angiogenic effects [18]. Furthermore, treatment of human ovarian microvascular endothelial cells with secretoneurin, the cleaved bioactive peptide of SCG2, significantly promoted ovarian angiogenesis by increasing endothelial cell migration [19]. In this study, the regulatory role of SCG2 in tumor angiogenesis in relation to cytokine excretion by tumor cells was investigated rather than solely focusing on the direct role of SCG in endothelial cells. The results revealed that ectopic expression of SCG2 in tumor cells significantly inhibited tumor angiogenesis in mice. Treatment with conditioned medium efficiently blocked the migration and sprouting of HUVEC. The conflicting role of SCG2 in angiogenesis demonstrated in the present and previous studies [18, 19] could be due to the use of different cell types. Nonetheless, the results of the present study provide multiple angles of evidence of the role of SCG2 in angiogenesis.

To clarify the molecular mechanism underlying the inhibitory effect of SCG2 on tumor angiogenesis, transcriptional profiling indicated that VEGF, which is a promoter of angiogenesis and is defined as a therapeutic target of tumor angiogenesis [10, 26, 27], was inhibited by SCG2 in cancer cells. Rescuing of VEGF in conditioned medium from SCG‐overexpressed cells efficiently blocked the SCG2‐induced inhibition of tumor angiogenesis. The results of this study also showed that expression of VEGF was inhibited by SCG2 at the mRNA and protein level, suggesting that SCG2 inhibits expression of VEGF at the transcriptional level. However, the location of SCG2 in the cytoplasm indicated that SCG2 may inhibit the transcription of VEGF by regulating the expression of transcription factors, such as HIF‐1 [28, 29, 30]. Also, SCG2 inhibited expression of HIF‐1α in cancer cells by interacting with VHL, a ubiquity‐degrading enzyme of HIF‐1α [31, 32, 33]. However, expression of VHL was not regulated by SCG2 in cancer cells, suggesting that SCG2 may promote the role of HIF‐1α in ubiquity degradation via binding VHL. Therefore, further studies are needed to clarify the mechanisms underlying this process.

## 5 Conclusion

In summary, the results of this study clarified the function and mechanism of SCG2 in the regulation of CRC tumor growth, which may provide a potential therapeutic target and a prognostic predictor. The identified role of SCG2 in tumor angiogenesis provided multiple angles of evidence of the role of SCG2 in angiogenesis. However, further studies are required to determine the potential role of SCG2 in CRC carcinogenesis in conditional knockout mice.

## Conflict of interest

The authors declare no conflict of interest.

## Author contributions

CF, LD, and CWF were responsible for the design and conduct of experiments, analysis, interpretation of data, and graphing. CW, LY, and YYY provided the survival data. CF drafted the manuscript. All authors read and approved the final manuscript. HXD and ZGZ supervised the whole analysis and provided guidance and instructions. Consent to publish has been obtained from all authors.

## Ethical approval

The relevant institutional Ethics Committees approved this study.

## Data accessibility

All data in our study are available upon request.

### Peer Review

The peer review history for this article is available at https://publons.com/publon/10.1002/1878‐0261.13044.

## Supporting information


**Fig. S1**. SCG2, HIF‐1α, VHL and hydroxyl‐HIF‐1α expression under hypoxia. (A) Proteins of the indicated cells after hypoxia exposure were extracted for western blot analysis. (B, C) The supernatants of the indicated cultured cells after hypoxia exposure were collected and used as conditioned medium for HUVEC tube formation. The branch points per frame were counted and analyzed (*n* = 4, ** *P* < 0.01). (D, E) The supernatant of the indicated cultured cells after hypoxia exposure was collected and used as conditioned medium for the HUVEC invasion assay. The invaded cells per frame were counted and analyzed (*n* = 4, ** *P* < 0.01).
