# Supplementary figures and images for: Secretogranin II impairs tumor growth and angiogenesis by promoting degradation of hypoxia‐inducible factor‐1α in colorectal cancer
Source: Mol Oncol. 2021 Jul 26;15(12):3513–26. doi: 10.1002/1878-0261.13044 (PMC8637574; doi:10.1002/1878-0261.13044)

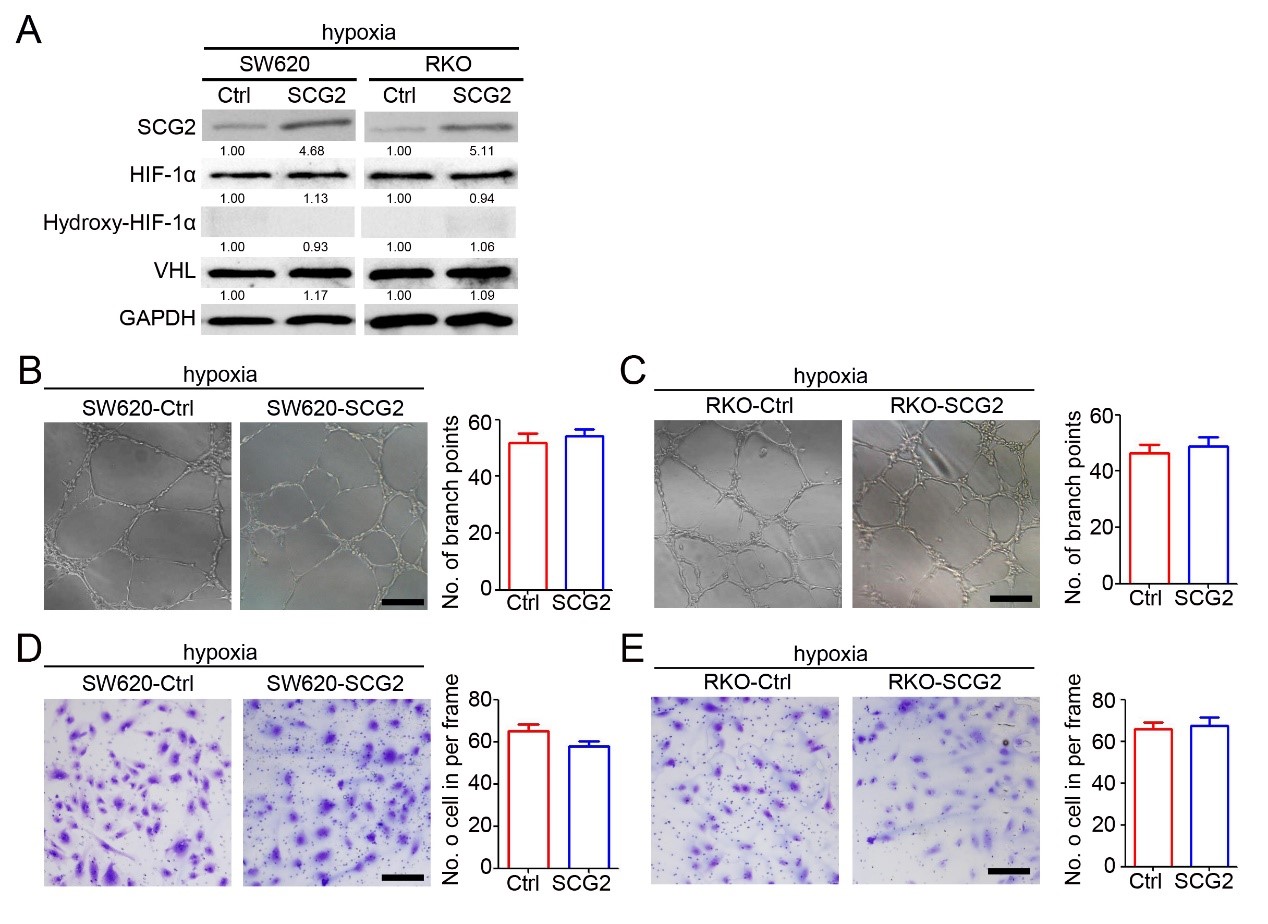

Supplement: Supplementary file 1 — Fig. S1. SCG2, HIF‐1α, VHL and hydroxyl‐HIF‐1α expression under hypoxia. (A) Proteins of the indicated cells after hypoxia exposure were extracted for western blot analysis. (B, C) The supernatants of the indicated cultured cells after hypoxia exposure were collected and used as conditioned medium for HUVEC tube formation. The branch points per frame were counted and analyzed (n = 4, ** P < 0.01). (D, E) The supernatant of the indicated cultured cells after hypoxia exposure was collected and used as conditioned medium for the HUVEC invasion assay. The invaded cells per frame were counted and analyzed (n = 4, ** P < 0.01). [file MOL2-15-3513-s001.jpg]
